# Epidermal growth factor receptor as a novel molecular target for aggressive papillary tumors in the middle ear and temporal bone

**DOI:** 10.18632/oncotarget.3605

**Published:** 2015-03-15

**Authors:** Shigeru Kawabata, M Christine Hollander, Jeeva P. Munasinghe, Lauren R. Brinster, José R. Mercado-Matos, Jie Li, Lucia Regales, William Pao, Pasi A. Jänne, Kwok-Kin Wong, John A. Butman, Russell R. Lonser, Marlan R. Hansen, Richard K. Gurgel, Alexander O. Vortmeyer, Phillip A. Dennis

**Affiliations:** ^1^ Department of Oncology, Johns Hopkins University School of Medicine, Baltimore, MD, USA; ^2^ Medical Oncology Branch, Center for Cancer Research (CCR), National Cancer Institute (NCI), National Institutes of Health (NIH), Bethesda, MD, USA; ^3^ Mouse Imaging Facility, National Institute of Neurological Disorders and Stroke (NINDS), NIH, Bethesda, MD, USA; ^4^ Division of Veterinary Resources, NIH, Bethesda, MD, USA; ^5^ Department of Pathology, Yale University School of Medicine, New Haven, CT, USA; ^6^ Memorial Sloan-Kettering Cancer Center, New York, USA; ^7^ Division of Hematology-Oncology, Department of Medicine, Vanderbilt-Ingram Cancer Center, Vanderbilt University School of Medicine, Nashville, TN, USA; ^8^ Department of Medical Oncology, Dana-Farber Cancer Institute, Boston, MA, USA; ^9^ Radiology and Imaging Sciences, Clinical Center, NIH, Bethesda, MD, USA; ^10^ Surgical Neurology Branch, NINDS, NIH, Bethesda, MD, USA; ^11^ Department of Otolaryngology-Head and Neck Surgery, University of Iowa Hospitals and Clinics, Iowa City, IA, USA; ^12^ Division of Otolaryngology-Head and Neck Surgery, University of Utah, Salt Lake City, UT, USA; ^13^ Present address: Laboratory of Cancer Biology and Genetics, CCR, NCI, Bethesda, MD, USA

**Keywords:** mouse model of adenomatous ear tumor, ear tumorigenesis, EGFR, EGFR-targeted therapy

## Abstract

Adenomatous tumors in the middle ear and temporal bone are rare but highly morbid because they are difficult to detect prior to the development of audiovestibular dysfunction. Complete resection is often disfiguring and difficult because of location and the late stage at diagnosis, so identification of molecular targets and effective therapies is needed. Here, we describe a new mouse model of aggressive papillary ear tumor that was serendipitously discovered during the generation of a mouse model for mutant EGFR-driven lung cancer. Although these mice did not develop lung tumors, 43% developed head tilt and circling behavior. Magnetic resonance imaging (MRI) scans showed bilateral ear tumors located in the tympanic cavity. These tumors expressed mutant EGFR as well as active downstream targets such as Akt, mTOR and ERK1/2. EGFR-directed therapies were highly effective in eradicating the tumors and correcting the vestibular defects, suggesting these tumors are addicted to EGFR. EGFR activation was also observed in human ear neoplasms, which provides clinical relevance for this mouse model and rationale to test EGFR-targeted therapies in these rare neoplasms.

## INTRODUCTION

Adenomatous tumors in the middle ear and temporal bone, especially mastoid and petrous portions, are rare [1]. Because of this rarity, they lack molecular characterization and effective chemotherapies. The anatomic and cellular origin of aggressive papillary tumors in the middle ear and temporal bone has not been clear. In 1989, Heffner proposed that temporal bone tumors that have low grade papillary adenocarcinomatous features originate in the endolymphatic sac [2]. Endolymphatic sac tumors (ELST) are defined as non-metastasizing adenocarcinomas of endolymphatic sac origin, but can often invade the petrous bone even though they are slow growing [1]. At early stages, ELSTs are typically confined within the region of the vestibular aqueduct, whereas later stage tumors destroy much of petrous bone including the apical part and invade into the middle ear [1, 3]. Although sporadic cases of ELST are commonly unilateral and occur with or without somatic mutations in the von Hippel-Lindau (*VHL*) gene [4, 5], bilateral ELSTs are a frequent manifestation of VHL disease [6]. Histopathologically, ELSTs are described as variable papillary-glandular adenocarcinomas of low histological grade, with cystic spaces and vascular papillae [7].

Although ELSTs show petrous temporal bone erosion, there are several reports that describe aggressive papillary tumors as filling the tympanic cavity and mastoid segment of the middle ear without involvement of the petrous temporal bone [3, 8, 9]. Tysome et al. reviewed 24 previous reported cases of aggressive papillary tumors in the middle ear and temporal bone. Twenty two cases showed invasion of the apical petrous temporal bone on imaging, suggesting that they arose from primary ELSTs. On the other hand, both in Tysome's case and the 2 others reported, there was no involvement of the petrous temporal bone at all, indicating the possibility that these aggressive papillary tumors that are confined to the middle ear and mastoid may be an entity separate from the ELSTs [3]. It remains controversial whether all aggressive papillary middle ear tumors are classified as ELSTs.

Surgical resection is the primary treatment of aggressive papillary tumors in the middle ear and temporal bone [10]. Early diagnosis is essential for a curative, complete excision. However, they are difficult to detect at an early stage and may present with severe audiovestibular or facial nerve dysfunction. In such cases, complete resection is often difficult because of the anatomic site and the late stage at diagnosis. The development of effective chemotherapies or targeted therapies as adjuvant or neoadjuvant treatments is desirable, because such treatments could shrink the tumor prior to surgery or treat patients with aggressive, unresectable tumors. This process would be expedited by the development of a mouse model that mimics human ear tumors and the identification of the molecular drivers in the mouse model and human ear tumors.

The epidermal growth factor receptor (EGFR) is a cell surface protein, and functions as a tyrosine kinase to transduce signals across the plasma membrane into the cytoplasm to promote cell growth and proliferation. Somatic mutations of *EGFR* in exon 21 (e.g., L858R) and exon 19 deletions have frequently been identified in patients with non-small cell lung cancer (NSCLC) who are frequently non-smokers [11]. These mutations are gain-of-function and enhance autophosphorylation of EGFR, which increases activation of downstream pathways such as the PI3K/Akt pathway and MEK/ERK pathway. EGFR-mutant lung cancers are highly sensitive to EGFR-specific tyrosine kinase inhibitors (TKIs) [12]. During the generation of a mouse model for mutant EGFR-driven lung cancer [13], we serendipitously discovered a new genetically engineered mouse (GEM) model of aggressive papillary ear tumor. EGFR-directed therapies corrected vestibular defects, induced ear tumor regression, and inhibited EGFR. Combined with the detection of active EGFR in human specimens of aggressive papillary ear tumors, these studies identify EGFR as a new molecular target for these rare ear neoplasms.

## RESULTS

### A new mouse model of aggressive papillary ear tumor

**Figure 1:**
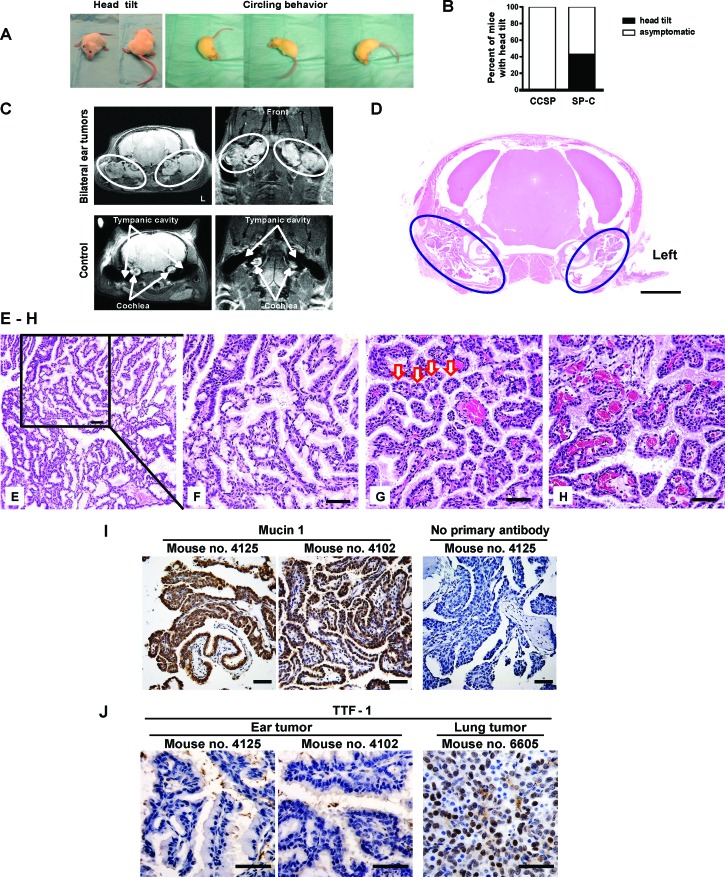
A new mouse model of aggressive papillary ear tumor (A) Vestibular dysfunction. Mouse no. 9498 (31.9-week-old) and no. 9492 (25.6-week-old) showed head tilt and circling behavior, respectively. See also Movie S1. (B) Head tilt of SP-C/mEGFR^L+T^ mice. Head tilt was evident in 43.0% (108/251) of SP-C/mEGFR^L+T^ mice (median age of the 251 mice: 30.6 weeks, range: 11.4-88.3 weeks). The median age of the 108 mice (male: 57, female: 51) with head tilt was 25.3 weeks (range: 11.4-55.1 weeks). (C) Bilateral ear tumors by MRI in a symptomatic SP-C/mEGFR^L+T^ mouse no. 9499 (31.9-week-old). Coronal (upper left) and horizontal (upper right) planes are shown. White ovals indicate dense areas of ear tumors. L, left side. A wild type asymptomatic mouse no. 9777 on FVB background was underwent MRI scan as a control. (D) Pathological diagnosis in a symptomatic SP-C/mEGFR^L+T^ mouse. A decalcified skull from mouse no. 9497 (22.9-week-old) with head tilt was assessed histopathologically. Hematoxylin and eosin (H&E) staining shows bilateral ear tumors in the tympanic cavity. Navy blue ovals indicate dense areas of ear tumors. The scale bars represent 2 mm. (E-H) Papillary adenocarcinomas in SP-C/mEGFR^L+T^ mice with head tilt. Representative H&E stainings of ear tumors from mouse no. 9496 (35.0-week-old, E and F) and no. 4103 (27.0-week-old, G and H). Red arrows in Figure 1G indicate acinar formations. The scale bars represent 50 μm. (I) Expression of Mucin 1 that is also referred to as EMA in ear tumors. Decalcified skulls from mouse no. 4125 (22.1-week-old) and no. 4102 (27.0-week-old) with head tilt were assessed histopathologically. IHC was performed as described in Supplementary Materials and Methods. The scale bars represent 50 μm. (J) Assessment of TTF-1 expression in ear tumors from mouse no. 4125 and no. 4102. As a control of TTF-1 positive staining, mEGFR^L+R^-driven lung tumor from C/L858R+T790M mouse no. 6605 treated with doxycycline was used. IHC was performed as described in Supplementary Materials and Methods. The scale bars represent 50 μm.

A human surfactant protein C (*SP-C*) was used as a lung-specific gene promoter to drive tetracycline-inducible expression of mutant EGFR^L858R+T790M^ (mEGFR^L+T^) [13]. After 10 weeks, a subset of SP-C/mEGFR^L+T^ mice exhibited head tilt with or without circling behavior, which was independent of doxycycline administration (Figure 1A, Movies S1 and S2, and Figure S1A). As the mouse colony expanded and this phenotype continued to be observed, doxycycline was omitted. At a median age 25.3 weeks (range: 11.4-55.1 weeks), 108 of 251 SP-C/mEGFR^L+T^ mice (43.0%) developed head tilt (Figure 1B). MRI scans showed bilateral ear tumors that were located in the tympanic cavity posterior to the cochlea (Figure 1C and Movies S3 and S4), which suggested the presence of tumors of the middle ear or ELSTs. Micro-computed tomography (CT) images of the chest from SP-C/mEGFR^L+T^ mice bearing ear tumors did not show lung tumors (Figure S1B), which was confirmed by autopsy and pathological analysis (data not shown).

Examination of a decalcified skull from a mouse with head tilt showed bilateral ear tumors in the tympanic cavity (Figure 1D). Histopathology revealed low-grade (i.e., differentiated) papillary adenomatous ear tumors that were characterized by papillomatous proliferation with a single layer of cuboidal-polygonal epithelial cells with a mucinous appearance in the apical portion of the cytoplasm Figures 1E and 1F), acinar formation (Figure 1G), and a highly vascularized stroma (Figure 1H). Mitotic were not seen, but the papillary tumors had invaded the nerve sheath outside of the tympanic cavity (Figure S1C), the cochlea, and the tympanic bulla (Figure S1D), which are features of malignant tumors (i.e., carcinoma). Mucin 1, also known as epithelial membrane antigen (EMA), was expressed in the papillary adenomatous ear tumors, indicating that these tumors are epithelial neoplasms (Figure 1I). On the other hand, thyroid transcription factor-1 (TTF-1 or NKx2.1) was not expressed in ear tumors, indicating that these tumors were not metastatic from lung or thyroid tissues (Figure 1J).

To clarify if these tumors originate from the endolymphatic duct/sac or epithelial cells in the tympanic cavity within the petrous portion of temporal bone, high resolution 3D-MRI scans of heads from asymptomatic SP-C/mEGFR^L+T^ mice were performed. As shown in Figure S2, endolymphatic ducts were clearly followed from the distal to proximal portion (Figures S2A to S2I) without evidence of ELST. Next, serial histological thin sections of ear tissues from asymptomatic SP-C/mEGFR^L+T^ mice were examined. Although ear tumors were identified in the tympanic cavity, there was no evidence of proliferating cells in the intraosseous part of the endolymphatic duct/sac (Figure S3). These data suggest that these epithelial ear neoplasms are primary aggressive papillary adenocarcinomas that likely originate from epithelial cells in the tympanic cavity, which cause vestibular dysfunction manifest as head tilt and circling behavior.

### Activation of EGFR and the downstream pathways in ear tumors

**Figure 2:**
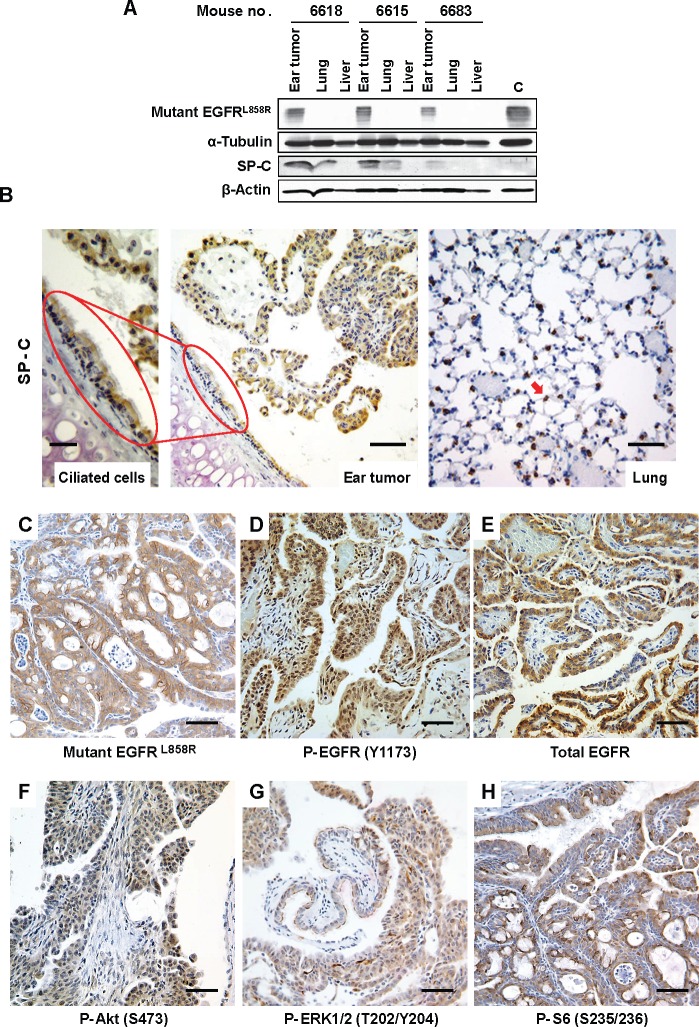
Activation of EGFR and the downstream pathways in ear tumors (A) Expression of mutant EGFR in ear tumors. Ear tumors, normal lung, and liver tissue were resected from 3 SP-C/mEGFR^L+T^ mice (32.3-38.3-week-old) that were administrated on NIH-31 regular diet. IB was performed using the indicated antibodies. C is a control that is mEGFR^L+R^-driven lung tumor lysate from C/L858R+T790M mouse treated with doxycycline. See also Figure S4. (B) SP-C expression in ciliated cells into the tympanic cavity and ear tumors. A decalcified skull from mouse no. 4125 was assessed histopathologically. IHC with anti-SP-C (FL-197 at 1:10,000) was performed as described in Supplementary Materials and Methods. Red arrow indicates an alveolar epithelial cell in the lung. The scale bar in left panel (Ciliated cells) represents 25 μm whereas the scale bars in middle and right panels (Ear tumor and Lung) represent 50 μm. (C-H) Activation of survival signaling, EGFR, Akt/mTOR, ERK1/2 pathways in ear tumors from mouse no. 4125. IHC was performed using the indicated antibodies. The scale bars represent 50 μm.

Because ear tumors developed in SP-C/mEGFR^L+T^ mice, but not in lung Clara-cell CCSP-driven mutant EGFR mice, we hypothesized that the expression of both SP-C and mEGFR^L+T^ might be involved in ear tumorigenesis. To test this hypothesis, we resected ear tumors macroscopically (Figure S4) and assessed expression of both proteins by immunoblotting. Mutant EGFR was selectively expressed in the ear tumors, whereas SP-C was expressed in both ear tumors and grossly normal lung tissue (Figure 2A), suggesting that SP-C-driven rtTA induces mEGFR^L+T^-driven tumorigenesis in the ear. To visualize SP-C expression in ear epithelium, immunohistochemical (IHC) analysis was performed. Even at an antibody dilution of 1:10,000, SP-C was clearly detected in ciliated cells in the tympanic cavity and ear tumors, as well as alveolar epithelial cells in the lung that served as a control (Figure 2B). These findings suggest that expression of SP-C in ear epithelium induces mEGFR^L+T^ protein, which causes ear tumorigenesis. Next, we evaluated activation of EGFR and downstream pathways in the ear tumors. IHC analysis showed expression of mutant EGFR in the tumors (Figure 2C). Increased phosphorylation of EGFR and downstream components such as Akt, mammalian target of rapamycin (mTOR) and extracellular signal regulated kinase1/2 (ERK1/2: classical mitogen-activated protein kinase1/2) was observed in tumors vs. surrounding stroma Figures 2D to 2H), suggesting that ear tumor development was dependent upon activation of EGFR and downstream pathways, and that EGFR inhibitors might be beneficial for ear tumor treatment.

### Regression of ear tumors by EGFR inhibitors

**Figure 3:**
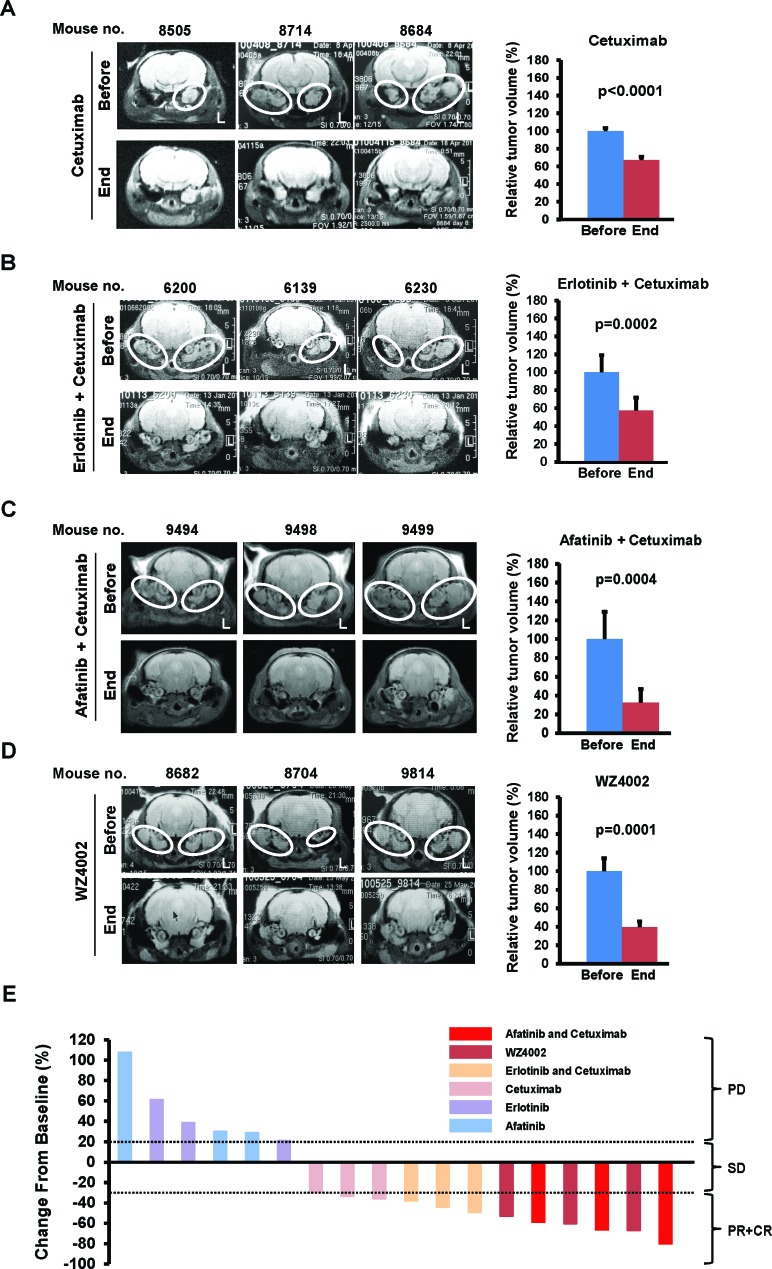
Regression of ear tumors by EGFR inhibitors (A-D) Assessment of ear tumor regression by MRI scan before and after treatment with EGFR inhibitors. SP-C/mEGFR^L+T^ mice with head tilt were randomized to 4 groups (3 mice/group) and then treated with cetuximab for 7 days (A), cetuximab combined with erlotinib or afatinib for 7 days (B and C), and WZ4002 for 5 days (D) as described dosing and schedule in Materials and Methods. See also Table S1. White ovals indicate dense areas of ear tumors. L, left side. *Columns*, mean of relative ear tumor volume from 3 mice in before and after treatment with each regimen. *bars*, standard deviation. Statistical analysis was performed with unpaired t test. P values of less than 0.05 were considered significant. (E) Change of tumor volume from baseline by EGFR inhibitors for individual SP-C/mEGFR^L+T^ mice bearing ear tumors in Table S1. CR, complete response; PR, partial response; SD, stable disease; PD, progressive disease. See also the criteria to classify tumor responses to drug treatment as described in Supplementary Materials and Methods.

Although mice with lung tumors driven by mEGFR^L+T^ are resistant to reversible EGFR TKIs used as single agents such as erlotinib or afatinib, combinations of EGFR-directed therapies can decrease expression of EGFR and tumor size [14]. Therefore, we performed a series of studies with the U.S. Food and Drug Administration (FDA)-approved EGFR inhibitors (erlotinib, a first generation TKI; afatinib, a second generation TKI; and cetuximab, an EGFR antibody) or an experimental third generation TKI (WZ4002) used singly or in combination (Table S1). Erlotinib or afatinib were ineffective as single agents (data not shown). Cetuximab alone decreased ear tumor volume by 32.9% (p< 0.0001; Figure 3A). The combination of cetuximab with erlotinib reduced tumor volume by 42.6% (p=0.0002; Figure 3B). Afatinib in combination with cetuximab reduced tumor volume by 67% (p=0.0004; Figure 3C) and normalized head tilt and gait as shown in Movies S1 (before treatment) and S5 (after treatment). These results indicate that FDA-approved agents such as cetuximab alone or combinations of cetuximab with FDA-approved EGFR TKI can reduce tumor volume and mitigate the phenotype in mice with mEGFR^L+T^-driven ear tumors.

Because WZ4002 can inhibit EGFR and shrink mEGFR^L+T^-driven lung tumors [13, 15], ear tumor-bearing mice were treated with WZ4002. These mice had a 60% reduction in ear tumor volume (p=0.0001; Figure 3D), with improvement in their circling behavior as shown in Movies S2 (before treatment) and S6 (after treatment), which is similar to the effects of afatinib combined with cetuximab. All mice treated with cetuximab and afatinib or erlotinib, or with WZ4002 alone, showed partial responses (PR), as well as 2/3 mice treated with cetuximab alone (Figure 3E and Table S1), which correlated with the inhibition of EGFR (Figure S5), whereas mice treated with afatinib or erlotinib alone showed progressive disease (PD). Taken together, these data indicate that ear tumors in SP-C/mEGFR^L+T^ mice are dependent upon EGFR activation, and that EGFR inhibitors can have pharmacodynamic effects in tumors that are located in the middle ear.

### Activated EGFR in human adenocarcinomas of the middle ear and ELSTs

**Figure 4:**
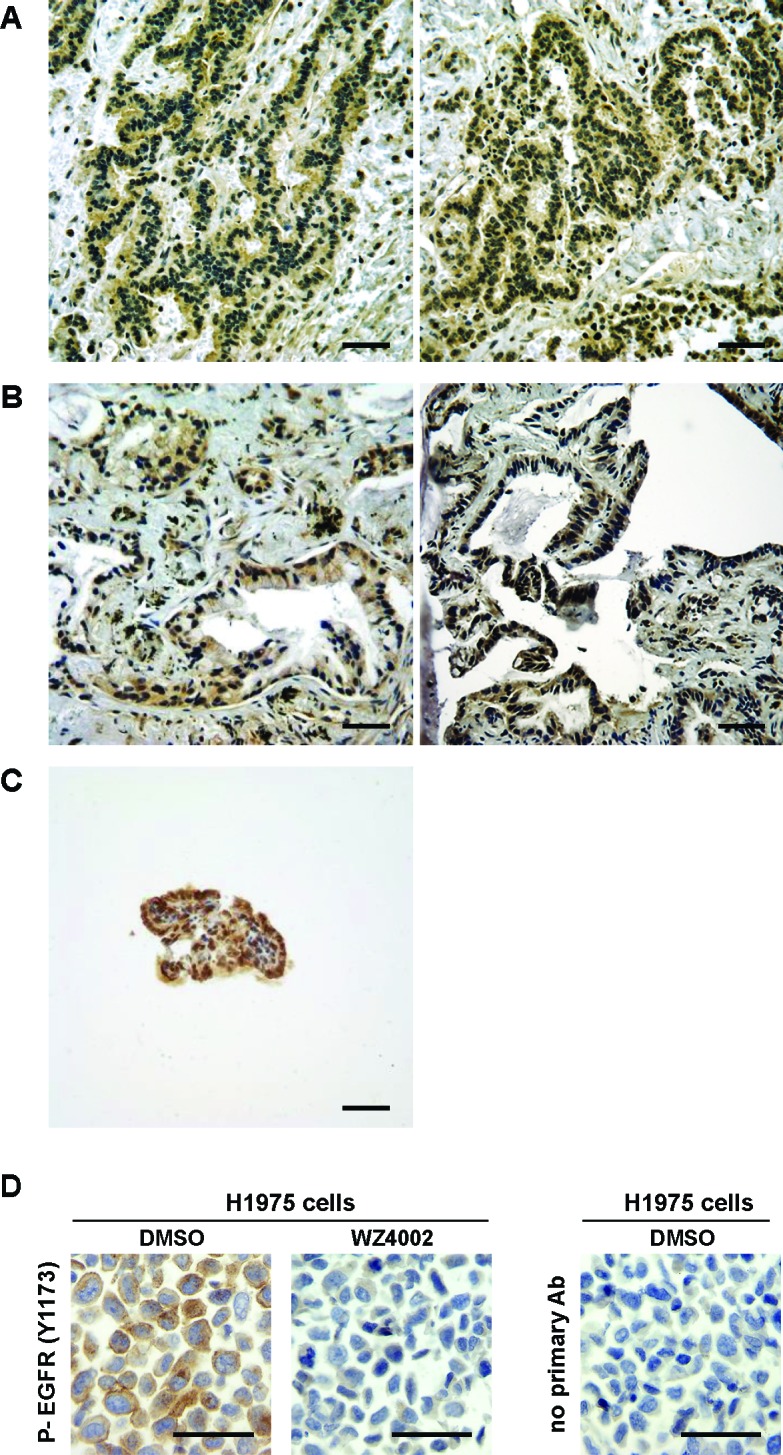
Activated EGFR in human adenocarcinomas of the middle ear and ELSTs (A-C) Photomicrographs show representative stainings for phosphorylated EGFR in resected specimens of low grade adenocarcinomas in left temporal bone from a patient #1 (A), adenocarcinomas with papillary structures in right tympanic cavity from a patient #2 (B), and ELSTs from a patient #3 with VHL disease (C). IHC was performed as described in Supplementary Materials and Methods. The scale bars represent 50 μm. (Note: 2 representative photomicrographs from one specimen of human adenocarcinomas are shown in Figures 4A and 4B). (D) EGFR assessment as a control of Figures 4A to 4C. Paraffin-embedded H1975 cell pellets treated with 0.5% DMSO or 1 μM WZ4002 for 16 hours were used as a control of the antibody specificity for IHC. The scale bars represent 50 μm. See also Supplementary Materials and Methods.

To assess the possible clinical relevance of our preclinical studies, activation of EGFR was assessed in human adenocarcinomas of the middle ear Figures 4A and 4B) and ELSTs (Figure 4C). IHC analysis was performed on formalin-fixed, paraffin embedded tissues with phospho-specific and native antibody against EGFR. Cell pellets from H1975 cells that have an EGFR T790M mutation were treated with WZ4002 or vehicle and used as controls (Figure 4D). These human adenocarcinomas of the middle ear and ELSTs displayed EGFR activation. Despite this activation, sequencing of exons 19-21 of *EGFR* in the 2 human adenocarcinomas of the middle ear did not reveal activating or resistance mutations (data not shown), suggesting other mechanism for EGFR activation. The detection of active EGFR in these specimens raises the possibility that EGFR-targeted therapies might have clinical efficacy in these rare ear neoplasms.

## DISCUSSION

Our studies identify EGFR as an oncogenic driver that initiates and maintains the neoplastic process in our mouse model, and is activated in human adenocarcinomas of the middle ear and ELSTs. Although we demonstrated that EGFR inhibitors can be effective in this model system, the fact that the mutant transgenic EGFR in this mouse model propagates EGFR activation to Akt, mTOR, and ERK1/2, suggests that inhibitors of these kinases might also have a role in these tumors. Thus, this mouse model could be used to assess different therapies to treat or prevent development of these ear tumors.

The SP-C/mEGFR^L+T^ mice model the human condition in several ways. First, mice become symptomatic due to vestibular dysfunction, which is observed in humans. Second, we found no evidence for metastasis of the murine ear tumors, which is similar to human tumors that rarely metastasize. Third, the cell of origin is unclear in our model, which mimics the controversy regarding cell of origin in human specimens. Finally, if additional oncogenic drivers are discovered in human ear tumor specimens, it is possible that relevant mouse models could be generated by using the SP-C promoter to drive expression of the human oncogenic driver.

The expression and activation of EGFR in this mouse model is probably controlled at different levels. For example, expression of mutant EGFR protein in ear tumors from SP-C/mEGFR^L+T^ mice was independent of doxycycline administration Figures 2A and 2C), indicating that this genetic system has leaky expression and/or that SP-C is expressed in ear epithelium. The leakiness of the SP-C promoter system was demonstrated by Perl et al., who assessed the inducibility of reporter gene expression in transgenic mice bearing *SP-C-rtTA* or *Ccsp-rtTA* ‘activator’ transgenes and a *tetO-luciferase* ‘target’ gene. Luciferase activity in the absence of doxycycline was only detected in SP-C-rtTA/tetO-Luc mice, but not CCSP-rtTA/tetO-Luc mice [16]. These findings were consistent with their other observations that doxycycline-independent expression of fibroblast growth factor-7 (FGF-7) was observed in SP-C-rtTA/tetO-FGF-7 mice [17]. Although leakiness is possible in this system, tumors were not observed in lung tissues or other tissues in the absence of doxycycline, suggesting that local expression of SP-C contributed to transgene induction. Indeed, we found that SP-C protein was expressed in ciliated cells of the mouse tympanic cavity and in ear tumors (Figure 2B), even though expression of SP-C was previously thought to be restricted to alveolar type II epithelial cells [18], Expression of SP-C in middle ear epithelium in our study is supported by the detection of *SP-C* mRNA in adult rabbit middle ear tissue [19]. Taken together, our results indicate that SP-C is expressed in not only mouse alveolar epithelial cells but also in middle ear epithelium, resulting in the induction of mutant EGFR and ear tumorigenesis.

How is EGFR activated in these middle ear tumors? Although this model is based on SP-C driven expression of a transgene with two different EGFR mutations (one that confers sensitivity to first generation EGFR TKI (L858R) and one that confers resistance (T790M)), it is not clear that either or both mutations are required to cause these tumors. Although other GEM models that utilize overexpression of EGFR or other mutations driven by SP-C might phenocopy this model, ear tumors have not been reported in other murine models of EGFR-driven lung tumors, including studies by Ohashi et al., who did not report ear tumor development in their SP-C L858R mice [20]. It is possible that the short latency of lung tumors induced by mutant EGFR in these studies may have masked development of ear tumors that would have occurred had the mice lived longer. Although activated EGFR was detected by IHC in human adenocarcinomas of the middle ear Figures 4A and 4B), we found no mutations in EGFR, suggesting that other mechanisms for EGFR activation. We were not able to perform gene copy analysis of *EGFR* because of the limited sample volume, but *EGFR* gene amplification is a potential mechanism for EGFR activation, because amplification and activation of EGFR has been reported in tumors from patients with glioblastoma multiform (GBM), NSCLC, and breast cancer [21, 22]. Human GBM xenograft tumors with amplification of wild-type *EGFR* show overexpression and higher activation of EGFR and invasive behavior compared to these without amplification [23]. Ligand dependent activation of EGFR could also contribute. For example, although the mechanism of action by which EGFR is activated in ELSTs from patients with VHL remains to be determined, loss of VHL increases levels of the EGFR ligand, TGF-α [21], and activates EGFR in renal cancer cells [24]. Similarly, silencing of *EGFR* decreases VHL-dependent renal cancer growth [25]. To assess the status of VHL in ear tumors from SP-C/mEGFR^L+T^ mice, we sequenced exons 1-3 in the mouse *Vhl* gene located on chromosome 6, and found no evidence of *Vhl* gene deficiency in these tumors (data not shown). These findings imply that a more comprehensive approach could identify the mechanisms by which EGFR is activated in human middle ear tumors, which could help direct therapeutic approaches.

Regardless of the mechanism by which it is activated, EGFR was effectively inhibited by a variety of EGFR-directed therapies in our studies, which improved the vestibular phenotype caused by the tumors. This has clinical implications. Although complete resection of aggressive papillary tumors of the middle ear or ELSTs can be curative [10], there is a need for medical therapies because the locally aggressive nature makes gross resection difficult without jeopardizing critical structures such as the inner ear, facial nerve, and great vessels at the skull base. Combining medical therapies with partial resection might allow for effective tumor control in patients with large tumors. In our preclinical studies, EGFR TKIs and/or an antibody against EGFR were effective against aggressive papillary adenocarcinomas that were located into the tympanic cavity and bulla, which correlated with inhibition of EGFR (Figure 3 and Figure S5). This indicates that EGFR-targeted therapies can be delivered to the complicated structures of the middle ear and temporal bones, raising the possibility that ineffective drug delivery might not limit human application of EGFR-targeted therapies for ear tumors. Our treatment studies showed that combinations of cetuximab with afatinib or erlotinib were superior to these single agents for the treatment of mEGFR^L+T^-driven ear tumors (Figure 3E), which is consistent with prior results for the treatment of mEGFR^L+T^-driven lung tumors [14]. Combining cetuximab with afatinib or erlotinib is well tolerated, and the maximum tolerated doses of these combinations have already been established in Phase I clinical trials [26, 27]. Moreover, our results confirmed that WZ4002, showed similar efficacy to the combination of cetuximab with afatinib Figures 3C and 3D). If further analysis of clinical ear tumor specimens shows non-mutational activation of EGFR, it is possible that EGFR inhibitors that were ineffective in our preclinical studies such as erlotinib or cetuximab might be effective clinically. Use of such drugs as single agents could mitigate toxicities associated with other EGFR inhibitors. The availability of multiple EGFR inhibitors for clinical trials might allow creative trial design that could incorporate novel sequences and combination of EGFR-directed therapies.

In summary, we characterized a novel mouse model and human ear tumor specimens to identify EGFR as a molecular target for aggressive ear tumors. The effectiveness of EGFR inhibitors in this mouse model supports clinical trials of EGFR inhibitors in humans with aggressive ear tumors. Given the readily availability of these FDA approved agents, EGFR inhibitors can be repurposed to meet an unmet medical need for these highly morbid but rare tumors.

## MATERIALS & METHODS

### Study design

The objective of this study was to develop a therapeutic drug for non-surgical therapy to human ear neoplasms. We performed preclinical studies with molecular targeted anticancer agents using an ear tumor mouse model, and evaluated a potential of molecular targeted therapy in human adenomatous ear tumors. First, we collected mice with head tilt or any sign of other vestibule dysfunction and then assessed ear lesions by radiological and histopathological approaches. Next the ear lesions were characterized by immunoblotting (IB) and immunohistochemical (IHC) analyses to develop a therapeutic drug. In a series of preclinical studies for ear tumor treatment with EGFR inhibitors, 3 mice bearing ear tumors were prepared for each regimen to do experimental replicates and analyze statistical data. The efficacy of regimen was assessed by radiological approach before and after each treatment, which was blinded to the investigators before measuring ear tumor volume. The primary end point was response rate assessed by criteria to classify tumor responses to drug treatment as described in Supplementary Materials and Methods. When mice showed partial response (PR) or progressive disease (PD), the treatment was stopped, and then the mice were euthanized for further analysis. Once a mouse showed severe vestibular dysfunction resulting in abnormal/reduced mobility and/or drug-induced toxicity (e.g., rapid weight loss or debilitating diarrhea), it was euthanized. To confirm clinical relevance of our preclinical studies in this project, a series of human ear neoplasms was analyzed for phosphorylation of EGFR, which validates our translational research.

### Mouse cohort and genotyping

All experiments were conducted under a protocol approved by the NCI animal care and use committee. NIH is an Association for Assessment and Accreditation of Laboratory Animal Care (AAALAC)-certified facility. The generation of transgenic progeny (tet-regulated *EGFR^L858R+T790M^* transgene; “L858R+T790M”) has been previously described [28]. Mice with *EGFR^L858R+T790M^* were subsequently crossed to surfactant protein C (*SP-C*)-reverse tetracycline responsive transactivator (*rtTA*) transgenic mice that were provided from Dr. Jeffrey A. Whitsett (University of Cincinnati College of Medicine, Cincinnati, OH, USA). Both transgenic mice were on an FVB background. *EGFR* and *SP-C-rtTA* genotype was assessed by PCR from tail clips [29, 30]. Bitransgenic mice harboring both *SP-C-rtTA* and *EGFR^L858R+T790M^* (SP-C/mEGFR^L+T^) were used in this ear tumor study. The generation of mouse lung-specific tumor model (harboring the *Clara cell secretory protein (Ccsp)-rtTA* and tet-regulated *EGFR^L858R+T790M^* transgenes; “C/L858R+T790M”) has been previously described [13, 28]. The expression of mEGFR^L+T^ was assessed by IB and IHC with an antibody to EGFR (L858R mutant specific, 43B2). All mice were housed in specific pathogen-free housing with NIH-31 regular diet and autoclaved water *ad libidum*.

### Ear tumor treatment study

Magnetic resonance imaging (MRI) scans were performed on SP-C/mEGFR^L+T^ mice with head tilt, and then ear tumor-bearing mice were treated with EGFR inhibitors. Afatinib and erlotinib were suspended in vehicle (0.5% Carboxymethylcellulose: Sigma C4888) and administrated by gavage at 20 mg/kg and 50 mg/kg once a day, daily for 7 days, respectively. Cetuximab (1 mg/mouse every 3 days for 7 days, i.e., day 1, 4, and 7) was injected intraperitoneally (i.p.) as described previously [14]. WZ4002 was formulated in vehicle (10% 1-Methyl-2-pyrrolidinone and 90% Polyethylene glycol-300 from Sigma, St. Louis, MO, USA) and administrated by gavage at 50 mg/kg twice a day, daily for 5 days as described previously [13]. Combining cetuximab with afatinib or erlotinib was administrated on each drug's schedule. All mice treated with EGFR inhibitors were rescanned by MRI to assess ear tumor regression. Response rate was evaluated by the criteria as described in Supplementary Materials and Methods. To examine the effects of EGFR inhibitors on biomarkers, ear tumor-bearing mice were given one dose of vehicle or combining cetuximab with afatinib or erlotinib, or given two doses of vehicle or WZ4002 using the same doses described above. Mouse skulls were extracted 3 hours after last administration. Mice and the weight were monitored daily while on study as well as diarrhea by visual inspection of fecal materials.

### Patients

Specimens of human ELSTs and adenocarcinomas of the middle ear were obtained from NINDS and University of Iowa Hospitals and Clinics, respectively. Information on gender, age, and histopathology was available for the samples. The approvals of the institutional review board of NINDS and University of Iowa Hospitals and Clinics were obtained for all studies.

### Statistical analysis

Statistical analysis was performed with unpaired t test. P values of less than 0.05 were considered significant. All analyses were performed using the GraphPad Prism software version 5.0c (GraphPad Software, Inc. La Jolla, CA, USA). The threshold value was set to 0.05.

## SUPPLEMENTARY MATERIALS, FIGURES, TABLES, MOVIES














