# Supplementary material for: Epidermal growth factor receptor as a novel molecular target for aggressive papillary tumors in the middle ear and temporal bone
Source: Oncotarget. 2015 Mar 15;6(13):11357–68. doi: 10.18632/oncotarget.3605 (PMC4484461; doi:10.18632/oncotarget.3605)
Supplement: Supplementary file 7 [file oncotarget-06-11357-s007.pdf]

Epidermal growth factor receptor as a novel molecular target for aggressive papillary tumors in the middle ear and temporal bone

Supplementary Material

Supporting Information file includes:

Supplementary materials and methods

Figure S1. A new mouse model of aggressive papillary ear tumor

Figure S2. Radiological assessment of the endolymphatic duct

Figure S3. Histological assessment of the origin of aggressive papillary ear tumor

Figure S4. Resected ear tumor from the tympanic cavity

Figure S5. Inactivation of EGFR in biomarker analysis

Table S1. Ear tumor treatment

References [28, 31, 32]

Movies S1 (Before treatment) and S5 (After treatment). Mouse no. 9498 treated with the combination of afatinib and cetuximab

Movies S2 (Before treatment) and S6 (After treatment). Mouse no. 8682 treated with WZ4002

Movies S3 (Coronal) and S4 (Horizontal). MRI scans of a head from mouse no. 9499

SUPPLEMENTARY MATERIALS & METHODS

Reagents

Afatinib and cetuximab were obtained from ChemieTek (Indianapolis, IN, USA) and Bristol-Myers Squibb (New York, NY, USA), respectively. WZ4002 compound was a kind gift from Dr. Nathanael S. Gray (Harvard Medical School, Boston, MA, USA). Erlotinib was obtained from LC Laboratories (Woburn, MA, USA). Primary antibodies for EGFR (L858R mutant specific, 43B2), P-EGFR (Tyr1173, 53A5), P-EGFR (Tyr1068, D7A5), EGFR, P-Akt (Ser473, D9E), P-p44/42 MAPK (Erk1/2) (Thr202/Tyr204, 20G11), P-S6 Ribosomal Protein (Ser235/236, D57.2.2E) were from Cell Signaling Technology (Beverly, MA, USA). Anti α-Tubulin and β-Actin (JLA20) antibodies were from Sigma (St. Louis, MO, USA) and Calbiochem (Gibbstown, NJ, USA), respectively. Anti Mucin 1 (H-295), TTF-1 (8G7G3/1), and SP-C (FL-197) antibodies were from Santa Cruz biotechnology (Santa Cruz, CA, USA).

Immunoblotting analysis (IB)

Cells (5×10^5 cells per well) were plated in six-well plates. The following day, cells were treated with WZ4002 or equal volume of DMSO for 16 hours and lysed in 2 x lysis buffer as described previously [31]. For tumor-tissue homogenates *in vivo*, frozen tumors were allowed to thaw on ice, then homogenized in radioimmunoprecipitation assay buffer [150 mmol/L NaCl, 1% Igepal CA-630, 0.5% sodium deoxycholate, 0.1% SDS, 50 mmol/L Tris (pH 8.0)] containing 2.5 mol/L h-glycerol phosphate, 0.2 mol/L sodium orthovanadate, 1.25 mol/L sodium fluoride, and 1 x protease inhibitor cocktail (Roche Diagnostics, Indianapolis, IN, USA) using a hand-held Tissue-Tearor homogenizer (Biospec Products, Bartlesville, OK, USA). Cell lysates or tumor-tissue homogenates with equal amounts of protein were separated by SDS-PAGE, and then

transferred to nitrocellulose membranes. The membranes were blocked for 1 hour in blocking buffer (1 x TBS, 5% milk, 0.1% Tween 20), and placed in primary antibody diluted in 1 x TBS, 5% bovine serum albumin, 0.1% Tween 20, overnight at 4°C. The following day, membranes were washed thrice in wash buffer (0.1% Tween 20, 1 x TBS). Primary antibody was detected using a horseradish peroxidase–linked secondary antibody, and visualized with the enhanced chemiluminescent detection system (Amersham Biosciences, Pittsburgh, PA, USA). Immunoblot experiments were performed at least 3 times.

Immunohistochemical analysis (IHC)

Expression of mutant $EGFR^{L858R+T790M}$ ($mEGFR^{L+T}$) was confirmed by IB and IHC with an antibody of EGFR (L858R mutant specific, 43B2). A formalin-fixed with decalcification, paraffin-embedded (FFPE) mouse skull was sectioned, placed on slide, and stained with hematoxylin and eosin (H&E) (Histoserv Inc. Germantown, MD, USA). Antigen retrieval was carried out using preheated target retrieval solution (pH 6.0) from DakoCytomation (Carpinteria, CA, USA) for 30 minutes in a boiling rice cooker. Vectastain Elite ABC kits from Vector Laboratories (Burlingame, CA, USA) were used according to manufacturer's instructions for blocking, dilution of primary antibody, and labeling. Primary antibody was incubated with sections for 16 hours at 4°C. 3,3- Diaminobenzidine was prepared fresh from tablets (Sigma). Specificity of staining was assessed by comparison with both samples stained in the absence of primary antibody and FFPE cell pellets from human non-small cell lung cancer (NSCLC) H1975 cells expressing $mEGFR^{L+T}$ protein.

Magnetic resonance imaging (MRI) Scanning and total tumor volume measurement

Anesthesia was induced then maintained with 1%-2% isoflurane administered via nosecone in an imaging chamber, and mice with vitals and body core temperature maintained were placed in a 72/25 mm volume/surface coil ensemble and positioned in a 7T horizontal MRI scanner. T_2 weighted anatomical images, encompassing the whole brain, were acquired in the coronal and horizontal planes (resolution= 75x75x750 µm, TR/TE= 2500/24 ms). For high resolution MRI, 3-Dimensional gradient-echo (GE) images (3D-MRI) of 0.2% gadolinium-perfused head were obtained at 14 T vertical scanner (TR/TE = 75/6 ms, isotropic resolution = 30x25x24 µm). The acquired images were viewed and analyzed using ImageJ version 1.43 software (http://rsbweb.nih.gov/ij/index.html). Ear tumor areas on the image sections were traced as regions of interest (ROIs). The areas of ROIs segmented with ImageJ were multiplied by the section thickness (750 µm), and then summed to obtain an estimate of the total tumor volume. Criteria to classify tumor responses to drug treatment were previously described [28] as the following: 1) complete response (CR): the disappearance of all target lesions; 2) partial response (PR): at least a 30% decrease in the volume of target lesions, taking as reference the baseline tumor volume; 3) progressive disease (PD): at least a 20% increase in the volume of target lesions, taking as reference the baseline tumor volume, and 4) stable disease (SD): neither sufficient shrinkage to qualify for PR nor sufficient increase to qualify for PD, taking as reference the baseline tumor volume.

Micro-computed tomography (CT) scanning

Anesthesia was induced then maintained with 1%-2% isoflurane administered via nosecone in an imaging chamber. Micro-CT scan was performed with an Inveon CT (Siemens Preclinical Solutions, Knoxville, TN, USA) using a 0.5 mm Al filter and 80 kVp X-rays with

380-uA current. The image acquisition was respiratory-gated. The isotropic resolution obtained was 43 μm. The resulting raw data were reconstructed to a final image volume of 480 X 480 X 480 slices at $(87\ \mu m)^3$ voxel dimensions using Siemens Inveon Acquisition Workplace version 1.4. The reconstructed images were viewed and analyzed using the ImageJ version 1.43 software.

Cell culture and Paraffin embedded cell pellets

H1975 human NSCLC cell line was a kind gift from Dr. William Pao (Vanderbilt-Ingram Cancer Center, Nashville, TN, USA). An activating somatic *EGFR* mutation (L858R) and a secondary *EGFR* mutation (T790M) have been detected in H1975 cells [32]. H1975 cells were maintained in 75 cm^2 flask in RPMI 1640 (Life Technologies, Grand Island, NY, USA) with 5% fetal bovine serum (FBS, Life Technologies) at 37°C in a 5.0% CO_2 atmosphere incubator. For FFPE H1975 cell pellets, cells (5×10^6 cells per flask) were plated in 15 cm plates. When the plates are approximately 80% confluent, cells were treated with 1 μM WZ4002 or equal volume of 0.5% DMSO for 16 hours and then harvested for fixing in 10% neutral buffered formalin. Formalin-fixed cells were sent to Histoserv Inc. and processed for FFPE pellets.

Sequencing of von Hippel-Lindau (*Vhl*) gene

For DNA preparation, the entire ear tumor pieces from SP-C/mEGFR^{L+T} mice were dissected and flash-frozen followed by stored at -70°C until being used. Identical weight of lung tissues from above mice were procured under identical procedures as internal controls for individual tumors. Tissues were chopped into <0.5 mm^3 pieces on dry ice and DNA was extracted with Qiagen DNAeasy Blood & Tissue kit (QIAGEN Inc., Valencia, CA, USA). In

PCR process, 6 pairs of primers were used to amplify the 2.7 kb fragment of *Vhl* gene. In brief, one pair was used for exon 1 and exon 2 amplification, respectively. Four pairs were used for exon 3 amplification; the overlapped 4 fragments (3a, 3b, 3c, 3d) cover the entire exon 3. Taq high-fidelity polymerase (Life Technologies, Grand Island, NY, USA) was used for PCR amplification, following below conditions: 95°C denaturation for 2 min, followed by 35 cycles of 95°C for 45 sec, 57°C for 45 sec, 72°C for 45 sec, followed by a 5min extension at 72°C. PCR products were visualized as single bands on agarose-gels and the bands were resected from the gel and DNAs were purified with Qiagen's Gel Extraction kit. PCR products were then cloned into Invitrogen's Topo10 vectors (Life Technologies) using the Topo-TA Cloning kit. After blue/white screening, 6 clones of each PCR product were prepared using Qiagen's DNA mini-prep kit. Cloning products were sequenced with Sanger's approach at W.M. Keck DNA Sequencing Facility at Yale University School of Medicine. Gene-specific primers (listed below) and vector's M13f/r primers were used for sequencing.

<u>For exon 1:</u>

Forward primer: 5' ATTTCCGCCTTCAAGTTGC 3'

Reverse primer: 5' CTTGTCCCTACCCACTGCAT 3'

<u>For exon 2:</u>

Forward primer: 5' GACCCGTGTGGCTTCTTACT 3'

Reverse primer: 5' TCTAACCCAGGTTGTCCTGG 3'

<u>For exon 3a:</u>

Forward primer: 5' TCTGCCTCTGAGGACTTGGT 3'

Reverse primer: 5' AAACGCTGGGACAGACCTCC 3'

<u>For exon 3b:</u>

Forward primer: 5’ CATCCCTGGGCTTTGTAGTG 3’

Reverse primer: 5’ TCCAGACAGTGGGTCACAGA 3’

For exon 3c:

Forward primer: 5’ AGCTCCACTCAGCCACAGAC 3’

Reverse primer: 5’ CCTTGAGTGCAGACTGATGC 3’

For exon 3d:

Forward primer: 5’ CCAGTGTGCTACCCTCCTTC 3’

Reverse primer: 5’ TACTGTGTTGTGTTACCTT 3’

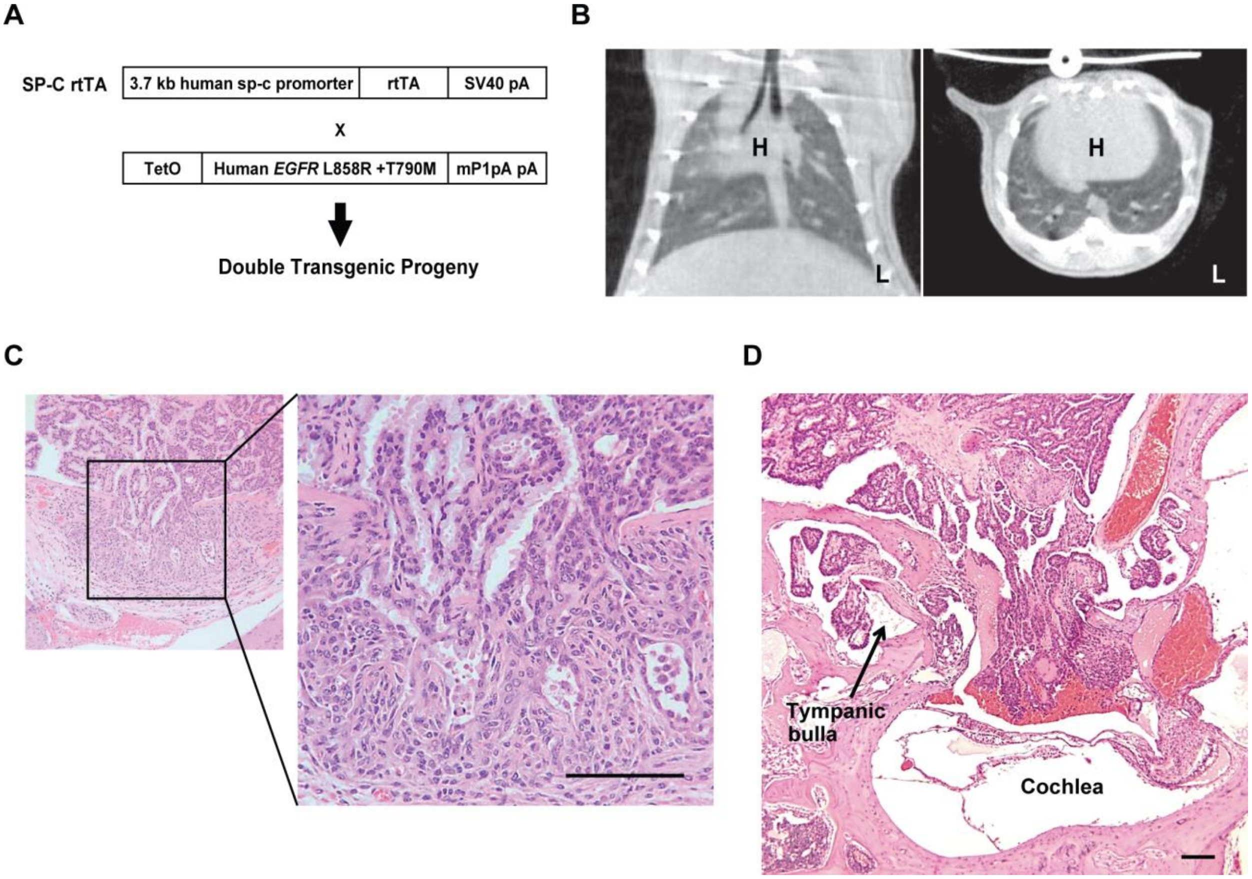

Figure S1. A new mouse model of aggressive papillary ear tumor

(A) Transgenic construct. Bitransgenic mice harboring both *SP-C-rtTA* and $EGFR^{L858R+T790M}$ were used in this study. **(B)** Representative micro-computed tomography (CT) images of mouse lung. Mouse no. 9492 (25.6-week-old) with bilateral ear tumors underwent micro-CT scan in the coronal (left) and transverse (right) planes to evaluate if it had lung tumor. H, heart; L, left side. **(C)** H&E stainings of ear tumors from mouse no. 4102 (27.0-week-old) show tumor invasion out of the tympanic cavity, which is the histopathological feature of malignant tumor (i.e., carcinoma). The scale bar represents 50 μm. **(D)** H&E staining of ear from mouse no. 9495 (22.9-week-old) shows that ear tumors in the tympanic cavity invade the cochlea and are located into the tympanic bulla. The scale bar represents 100 μm.

A - I

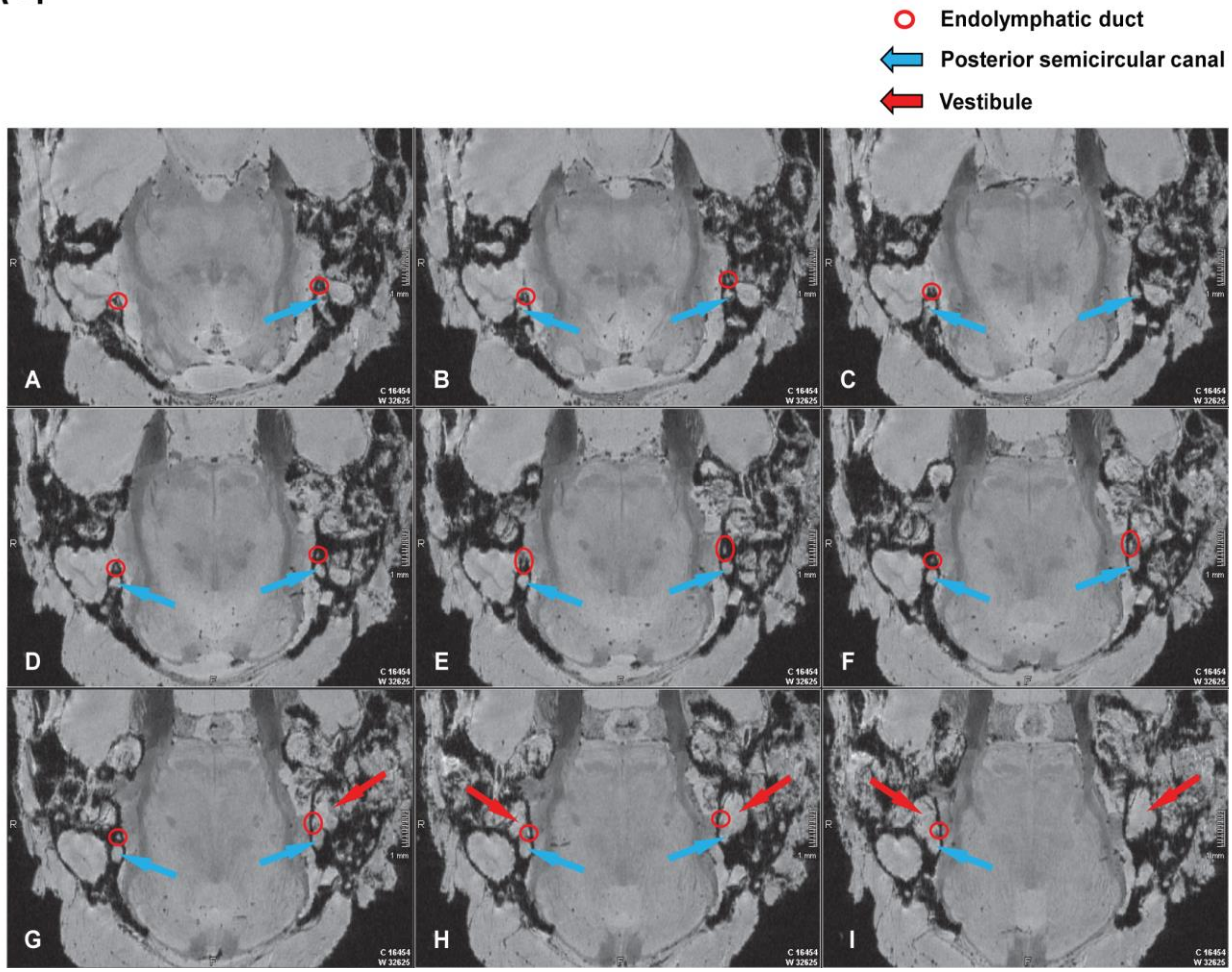

Figure S2. Radiological assessment of the endolymphatic duct

(A-I) High resolution 3D-MRI of a head from asymptomatic mouse no. 3441 (20.3-week-old). 3D-MRI scanning was performed as described in Supplementary materials and methods. Red circles and ovals indicate the endolymphatic duct. Blue and red arrows indicate the posterior semicircular canal and vestibule, respectively.

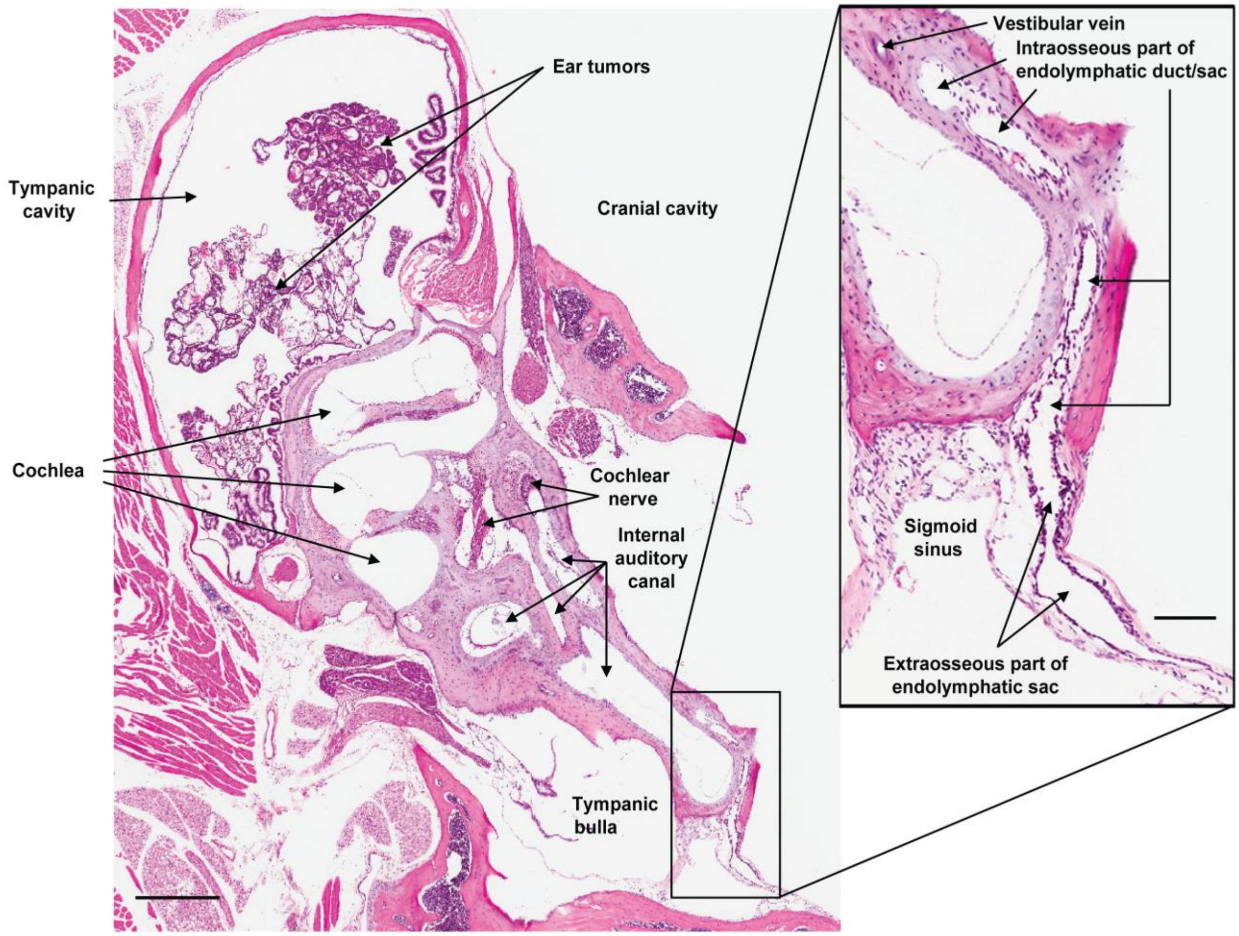

Figure S3. Histological assessment of the origin of aggressive papillary ear tumor

Representative H&E staining of 5 µm thin slice (sagittal section) of left ear from an asymptomatic mouse no. 3441 shows ear construct including the intraosseous and extraosseous parts of the endolymphatic duct/sac. The scale bars represent 500 µm (low magnification, left) and 200 µm (high magnification, right).

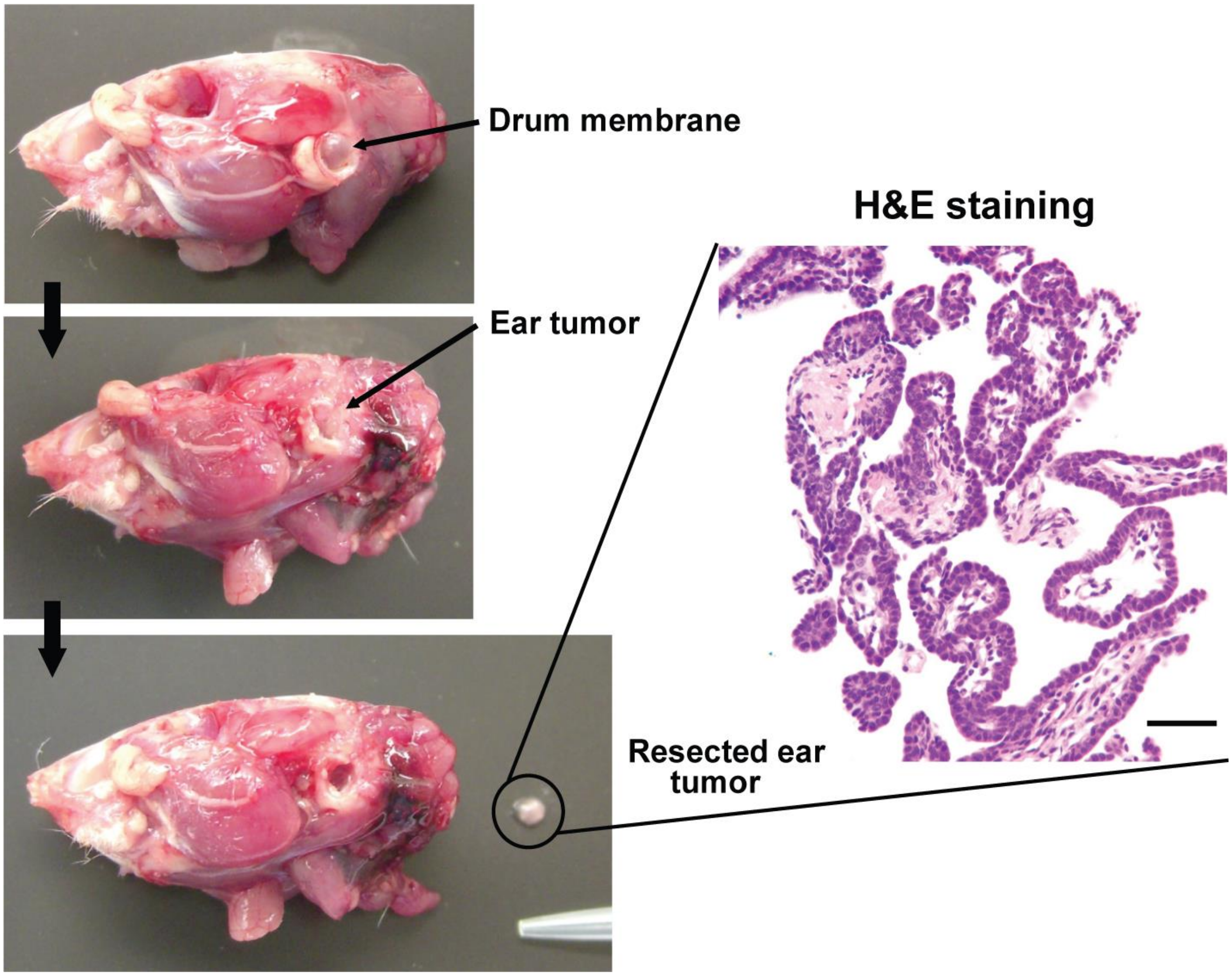

Figure S4. Resected ear tumor from the tympanic cavity

Bilateral ears of mouse no. 6234 (13.0-week-old) with head tilt were evaluated if there might be macroscopically-visible lesion into the tympanic cavity after removing the drum membrane. Resected lesion was assessed histopathologically. H&E staining shows epithelial ear neoplasms. The scale bar represents 50 μm.

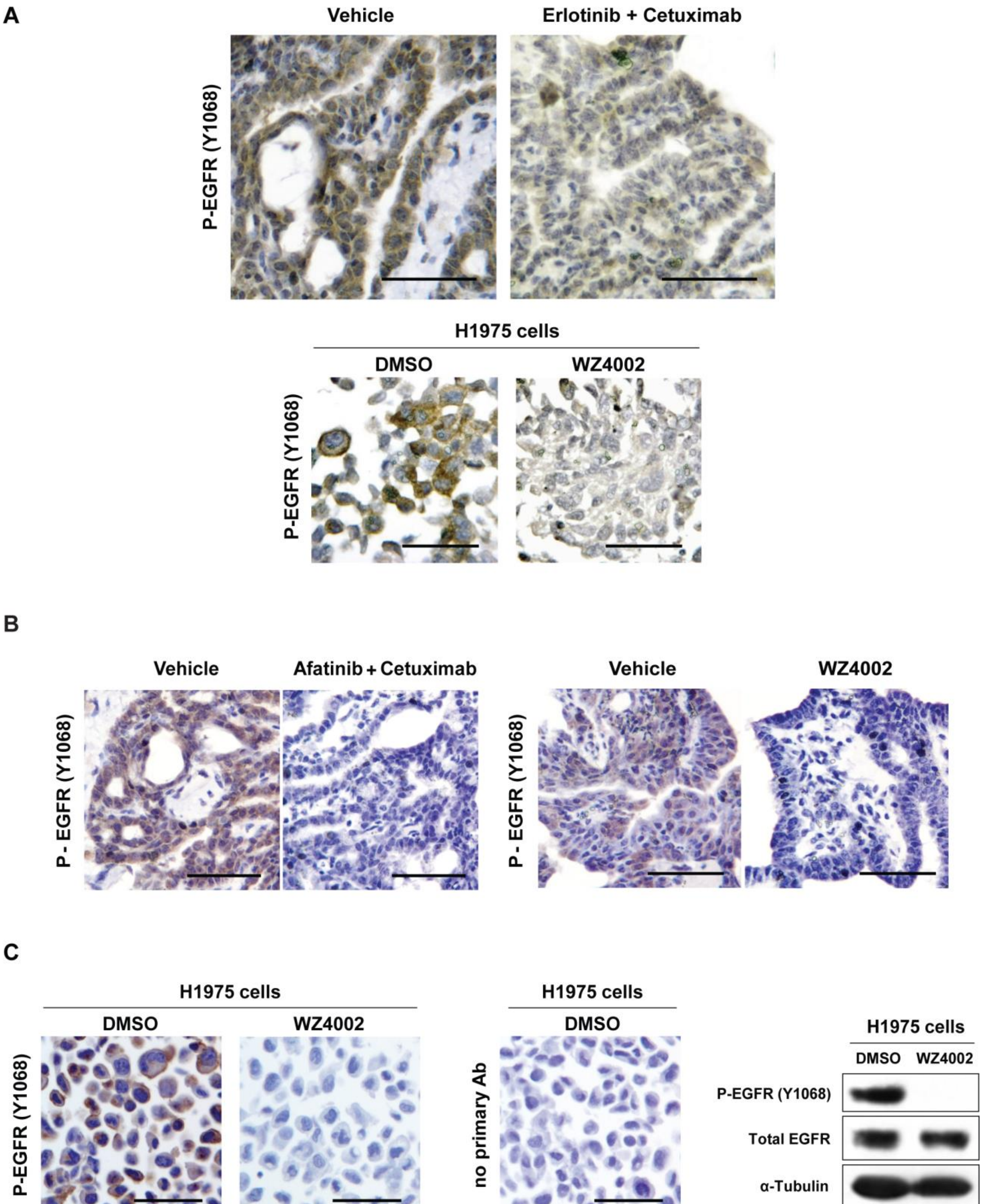
A
Vehicle
Erlotinib + Cetuximab
P-EGFR (Y1068)
H1975 cells
DMSO
WZ4002
P-EGFR (Y1068)
B
Vehicle
Afatinib + Cetuximab
Vehicle
WZ4002
P- EGFR (Y1068)
P- EGFR (Y1068)
C
H1975 cells
DMSO
WZ4002
P-EGFR (Y1068)
H1975 cells
DMSO
no primary Ab
H1975 cells
DMSO
WZ4002
P-EGFR (Y1068)
Total EGFR
α-Tubulin

Figure S5. Inactivation of EGFR in biomarker analysis

(A) EGFR inhibition by the combination of erlotinib and cetuximab. Six SP-C/mEGFR^{L+T} mice bearing ear tumors were given one dose of vehicle or the combination using the same doses described in Materials and methods. The skulls of mice were extracted 3 hours after last administration. Photomicrographs show representative stainings for phosphorylated EGFR in ear tumors treated with vehicle or the combination of erlotinib and cetuximab (upper). Paraffin-embedded H1975 cell pellets treated with 0.5% DMSO or 1 µM WZ4002 for 16 hours were used as a control of the antibody specificity for IHC (lower). IHC was performed as described in Supplementary materials and methods. The scale bars represent 50 µm. See also Figure 3B. **(B)** EGFR inhibition by the combination of afatinib and cetuximab or by WZ4002. Twelve SP-C/mEGFR^{L+T} mice bearing ear tumors were prepared for the assessment of EGFR activation. Six mice were given one dose of vehicle (0.5% Carboxymethylcellulose) or the combination of afatinib and cetuximab, whereas the other 6 mice were given two doses of vehicle (10% 1-Methyl-2-pyrrolidinone and 90% Polyethylene glycol-300) or WZ4002 using the same doses described in Materials and methods. The skulls of mice were extracted 3 hours after last administration. Photomicrographs show representative stainings for phosphorylated EGFR in ear tumors treated with vehicle or EGFR inhibitors. IHC was performed as described in Supplementary materials and methods. The scale bars represent 50 µm. See also Figures 3C and 3D. **(C)** Paraffin-embedded H1975 cell pellets treated with 0.5% DMSO or 1 µM WZ4002 for 16 hours were used as a control of the antibody specificity for IHC in Figure S5B. The decreased-EGFR phosphorylation by WZ4002 in H1975 cells was reconfirmed by IB (right). The scale bars represent 50 µm.

Table S1. Ear tumor treatment

Animal	Sex	Age (weeks) at Head tilt	Regimen	TV (mm3) Before therapy	TV (mm3) End therapy	Tumor Reduction by therapy (%)	Response to therapy
**9494**	m	30.1	Afatinib+Cetuximab	37.79	12.67	-66.47	PR
**9498**	m	31.9	Afatinib+Cetuximab	77.70	15.41	-80.17	PR
**9499**	f	31.9	Afatinib+Cetuximab	115.28	47.21	-59.05	PR
**8682**	m	17.9	WZ4002	79.48	26.19	-67.05	PR
**8704**	m	21.6	WZ4002	48.65	19.22	-60.50	PR
**9814**	m	39.9	WZ4002	71.06	33.30	-53.14	PR
**9492**	m	25.6	Afatinib	35.84	46.14	28.73	PD
**8778**	m	15.4	Afatinib	13.59	28.27	107.96	PD
**8791**	m	40.9	Afatinib	21.04	27.37	30.09	PD
**8505**	f	20.3	Cetuximab	34.11	21.89	-35.83	PR
**8714**	m	14.0	Cetuximab	38.12	26.76	-29.81	SD
**8684**	f	15.7	Cetuximab	37.38	24.94	-33.30	PR
**8811**	f	50.6	Erlotinib	29.96	48.29	61.16	PD
**6164**	m	33.4	Erlotinib	27.30	33.09	21.21	PD
**6145**	f	37.1	Erlotinib	32.53	45.09	38.62	PD
**6200**	m	28.7	Erlotinib+Cetuximab	48.84	30.35	-37.86	PR
**6139**	f	33.9	Erlotinib+Cetuximab	27.97	14.16	-49.37	PR
**6230**	f	25.6	Erlotinib+Cetuximab	29.34	16.43	-44.02	PR

TV, tumor volume; PR, partial response; SD, stable disease; PD, progressive disease. See also the criteria to classify tumor responses to drug treatment as described in Supplementary Methods.
